# A multivariate multilevel analysis of the risk factors associated with anthropometric indices in Iranian mid-adolescents

**DOI:** 10.1186/s12887-020-02104-x

**Published:** 2020-05-02

**Authors:** Marzieh Alamolhoda, Seyyed Taghi Heydari, Seyyed Mohammad Taghi Ayatollahi, Reza Tabrizi, Maryam Akbari, Arash Ardalan

**Affiliations:** 1 Health Policy Research Center, Institute of Health, Shiraz University of Medical Sciences, Shiraz, Iran; 2 Department of Biostatistics, Medical School, Shiraz University of Medical Sciences, Shiraz, Iran; 3 Department of Mathematics, Yasouj University, Yasouj, Iran

**Keywords:** Childhood obesity, Skinfold thickness, Socio-economic status, Physical activity, Family history of obesity, Multivariate multilevel analysis

## Abstract

**Background:**

The present study was conducted to jointly assess some specific factors related to body fat measures using a multivariate multilevel analysis in a representative sample of Iranian mid-adolescents.

**Methods:**

This study was conducted among 2538 students (1286 boys) aged 14–20 years old, who were randomly selected among 16 public high schools by multi-stage random sampling procedure from all education districts of Shiraz, Iran. Data on demographic characteristics, family history of obesity, physical activity, socio-economic (SES) variables and screen time were collected. Height, weight, triceps (TST), abdominal (AST), and subscapular (SST) skinfold thickness were measured and their body mass index (BMI) was calculated. A multivariate multilevel approach was used to analyze the factors associated with obesity measures of the TST, AST, SST at the child and district levels.

**Results:**

In this study, the prevalence of overweight and obesity was estimated to be 10.2 and 5.1%, respectively. Overall, the major portion of the total variance in TST (97.1%), AST (97.7%), and SST (97.5%) was found at the child level. The results of multivariate multilevel method revealed that being girls, having a family history of obesity, and SES were significantly associated with increasing of three body fat measures (all the *p*-values were less than 0.05). There were significant positive associations between moderate to vigorous physical activities with AST and SST (for AST: β =2.54, SE = 1.40, *p* = 0.05; for SST: β =2.24, SE = 1.20, p = 0.05). Compared to children in 14–16 age group, children in age group 16–18 years had less TST (β = − 0.67, SE = 0.34, *p* = 0.04). Furthermore, other age groups and screen time did not play an important role in three outcome variables.

**Conclusions:**

The results showed some factors that contribute to three body fat measures. Therefore, it is necessary to develop effective interventions to prevent the effects of individual and environmental undesirable factors on childhood obesity in both family and community levels.

## Background

In recent years, the rapid growth of obesity among children and adolescents has become a serious public health challenge in both developing and developed countries [1–3]. The prevalence of childhood obesity has an arising trend in Iran, like other developing countries [4, 5]. Obesity in early life, as an important metabolic problem leads to major health disorders such as hypertension [4], non-alcoholic fatty liver disease [6], obesity in the adulthood and more other nutrition -related chronic diseases such as type 1 diabetes, cardiovascular disease [7], some types of cancer [8] as well as a decrease in the life expectancy [9].

Among several approaches used to measure the obesity, Body Mass Index (BMI), Skinfold Thickness (ST) and waist circumference (WC) have been more frequently used in clinical setting [8]. Although, BMI, as a simple and inexpensive parameter is used more than other approaches for measuring the obesity, it has several drawbacks as mentioned in the literature [10]. ST is an easily obtained adiposity index, which is commonly used, and is an accurate estimate for measuring the subcutaneous body fat among children and adolescents [7, 11–13]. It also can be easily applied in clinics, laboratories and schools because of its portable, low cost and non-invasive nature [14]. Further, the use of ST as an epidemiological screening tool for cardio metabolic risk factors, a better predictor of high body fatness during adulthood than BMI and a reliable tool in assessing the effect of lifestyle factors in children and adolescent has been reported in earlier reports [15–17].

The mechanism of obesity development has remained unidentified, and the researchers characterize the obesity as a health disorder with multiple causes [18]. Certainly, a lot of influential factors have been reported to be effective on childhood obesity. Individual factors such as physical and social functioning as well as environmental factors, lifestyle preferences, and cultural environment play an important role in increasing or decreasing the prevalence of childhood obesity [19, 20]. A systematic review of the published studies in South Asian countries revealed that the lack of proper physical activities, prolonged TV watching or using different electronic media, unhealthy dietary patterns, family history of obesity, and the family socio-economic status are among the main individual factors found to be significantly associated with the obesity in children and adolescents [21]. Moreover, previous studies showed that behavioral and environmental factors were significantly associated with increasing childhood obesity [22, 23]. In fact, factors related to childhood obesity are a subset of multi-factorial etiology in three levels: family, school, and community. Therefore, the coverage of the risk factors contributing to childhood obesity needs to consider muti-sectoral approaches. However, many studies have examined simple relationships between predictor variables with adiposity indices and there are limited studies that have considered hierarchical structure in these models [19, 20, 24, 25]. It is necessary to consider effective strategies in order to prevent and control childhood obesity in different aspects.

Since anthropometric measures seemingly share common biological and environmental relationships, simultaneous evaluation of multiple outcomes and the influential covariates using multivariate multilevel approaches will lead to more accurate results than univariate approaches. Furthermore, when the data have a hierarchical structure, predictor variables in ordinary multivariate regression models with single level do not provide correct inferences for outcome variables, due to the dependency existing between the observations. Therefore, it is necessary to fit a model that can accurately estimate the parameters. The present study aimed to simultaneously investigate the relationship between the influential covariates and three anthropometric measures including triceps (TST), abdominal (AST), and sub-scapular (SST) skinfold thickness using multivariate multilevel analysis.

## Methods

### Subjects, study design, and sampling procedure

The sample of the current study was collected from high school students in Shiraz during September to December 2014. Administratively, Shiraz, the capital of Fars Province in southern Iran, is divided into 4 educational districts. Each district has distinct social, cultural, economic and health characteristics. In this cross-sectional study, 2538 healthy subjects (1286 boys and 1252 girls) aged 14–20 years old were selected among 16 public high schools by multi-stage random sampling procedure from 4 education districts of Shiraz. In the first step, 4 schools were chosen from each district (two from boy’s schools and two from girl’s schools) using simple random sampling. In the next step, based on the school sample size, 2 or 3 classrooms were randomly selected from each school, and all the students in the classroom were studied.

Children gave oral assent before participating in the study and written informed consent was obtained from their parents. The study protocol was approved by the Ethics Committee of Shiraz University of Medical Sciences. Moreover, the permission was obtained from schools̓ principal for collecting the data from the selected classrooms.

### Measurements

The collected data were classified into two groups: demographic characteristics and anthropometric measurements; the former describing sex, age, screen time, family history of obesity, Physical Activities (PA), and Socio-Economic Status (SES) variables. These data were collected through a questionnaire. Content validity of the questionnaire was confirmed by three specialists in epidemiology, biostatistics and endocrinology.

Screen time was defined as the times spent on watching TV, using computer, and playing video games by using a question: “How long do you spend your time on watching TV, using computer, and playing video game per day?”. Family history of obesity was assessed using a question, “Is there a history of obesity in your family?”. PA was assessed using two questions: during the past week, “What kind of physical activity do you do? “ and “How many days do you have physical activity for more than 30 min?”. PA was classified into three levels, namely mild, moderate, and vigorous activities. SES was calculated using principal component analysis by available variables used for SES measurement [19, 26]. Variables such as parents’ education level, parents’ occupation, as well as choice of car type and homeownership (Ownership or Rent) were included in the analysis to make one main component. The SES score calculated using the weighted averages of the variables was categorized into three levels (low, middle, and high) to define the SES.

The second data related to anthropometric measurements included body weight and height, BMI, TST, AST and SST. Height and weight were measured in all students, while wearing light clothing and no shoes, with 0.1 cm and 0.1 kg accuracy, respectively using tape measure and a SECA digital scale (Germany). BMI was calculated by dividing weight (kg) by height squared (m^2^) and was classified based on the WHOs̓ growth charts [27]. The subjects of the same sex and age with BMI less than 85th percentile, between 85th and 95th percentile and above 95th percentile were classified into three groups: normal, overweight and obese, respectively [28]. A graded caliper was used to measure the ST in three sites of the body (triceps, abdominal, and subscapular). To measure the triceps, the technician bent the elbow to 90 degrees and marked the point midway between the top of the shoulder and elbow, and then measured a vertical fold by the caliper at a 90-degree angle on that midway point with the arm hanging naturally at the subject’s side. For AST measurements, vertical folds were measured at 2 cm to the right and left of the navel. Finally, a diagonal fold (calipers held at a 45-degree angle) was taken across the back, just below the shoulder blade to measure the SST. ST was measured on both right and left sides of the body separately, and the average of two measurements was recorded to the nearest 0.5 mm [29]. All anthropometric measurements of the students were done by two trained technicians. Measurement was repeated by another technician if there was a great difference in the right and left sides.

### Statistical analysis

**Fig. 1:**
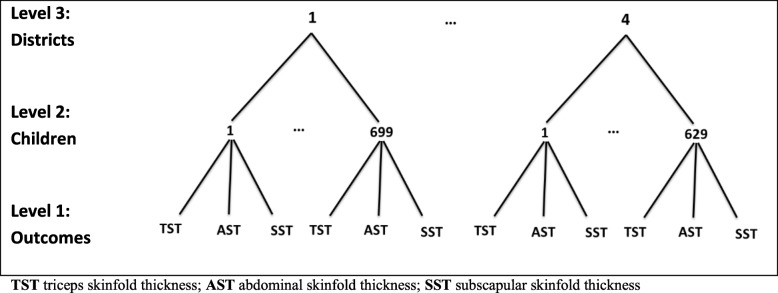
Multivariate multilevel structures of anthropometric measures (TST, AST and SST) at level one nested within children at level 2, nested within districts at level 3

Mean and standard deviation were calculated for quantitative data, and frequency and percentage were reported for qualitative variables. Pearson Chi-Square, and One-Way ANOVA tests were used to investigate the association between the variables at the child level. A *P*-value of less than 0.05 was considered as statistically significant. Since, in this study, the data had a hierarchical structure with multiple outcomes, multivariate multilevel analysis was used to depict the hierarchical structure of the data [30]. The ability to model the correlation between response variables (in our case, at individual and district levels), increasing the power, performing a single test to avoid the risk of chance capitalization, which is inherent to carrying out a separate test for each dependent variable, and measuring the effect of any exploratory variable separately across multiple outcome variables are main advantages using multivariate hierarchical analysis [31]. In this study, TST, AST, and SST as three multiple outcome variables were at the first level in the hierarchy. Therefore, for each subject, three quantitative measures were recorded simultaneously as units in level 1. The subjects included as units in the second level, and districts were considered at the third level in the hierarchy. These levels are shown in Fig. 1. The multilevel structure makes it possible to evaluate whether the districts made a difference to individual anthropometric measures. Three outcome variables were regressed on a set of explanatory variables in the random intercept model, which were in levels 2 and 3. Primary analysis of the data was carried out using SPSS software (Ver. 18.0). The MLwiN software version 2.00 was used to analyze the hierarchical model.

## Results

Table 1 shows the results of descriptive statistics (percentage) for the children in 4 districts. A total of 1286 (50.7%) subjects were boys and were roughly distributed equally in 4 districts. Mean age (SD) of the participants was equal to 15.99 (0.94) years old, which was not distributed equally in the districts (*P*-value < 0.05). The distribution of screen time was somewhat different between 4 districts (*p* = 0.05). On average, more times on watching TV or using computer were recorded for students living in districts 4 and 1 (Means (SD) in 4 districts were 4.47 (2.29), 4.39 (2.12), 4.36 (2.52) and 4.72 (2.52) h/day, respectively). About 44% of the participants were categorized into family history of obesity group. Having mild physical activities was reported by 76.6% of the students and only 3.1% of them had vigorous physical activities. Compared to other districts, more children from district 3 lived in a family with low SES. The results of Chi-Square test revealed that, the subjects were distributed differently with regard to physical activities, SES and prevalence of overweight and obesity in four districts. However, there was not a statistically significant difference between the four groups with respect to gender and family history of obesity.

**Table 1:** Descriptive statistics among individuals by districts

Variables	Descriptor	District 1 (*n* = 699)	District 2 (*n* = 601)	District 3 (*n* = 609)	District 4 (*n* = 629)	Total (*n* = 2538)	*P*-value^a^
**Screen time**							
< 2 h/day	N (%)	51 (9.5)	43 (8.5)	48 (13.7)	43 (8.7)	185 (9.8)	0.05
≥ 2 h/day	N (%)	486 (90.5)	463 (91.5)	302 (86.3)	452 (91.3)	1703 (90.2)	
**Sex**							
Boy	N (%)	359 (51.4)	309 (51.4)	295 (48.4)	323 (51.4)	1286 (50.7)	0.66
Girl	N (%)	340 (48.6)	292 (48.6)	314 (51.6)	306 (48.6)	1252 (49.3)	
**Age groups (year)**							
[14–16)	N (%)	362 (51.9)	347 (57.8)	320 (52.6)	347 (55.2)	1376 (54.3)	0.00
[16–18)	N (%)	328 (47.0)	252 (42.0)	254 (41.8)	260 (41.3)	1094 (43.2)	
[18–20]	N (%)	8 (1.1)	1 (0.2)	34 (5.6)	22 (3.5)	65 (2.6)	
**Family history of obesity**							
Yes	N (%)	296 (42.6)	261 (43.8)	270 (44.6)	286 (45.9)	1113 (44.2)	0.67
No	N (%)	399 (57.4)	335 (56.2)	336 (55.4)	337 (54.1)	1407 (55.8)	
**Physical activity**							
Mild	N (%)	431 (75.0)	315 (72.9)	355 (82.2)	379 (76.7)	1480 (76.6)	0.02
Moderate	N (%)	124 (21.6)	99 (22.9)	65 (15.0)	105 (21.3)	393 (20.3)	
Vigorous	N (%)	20 (3.5)	18 (4.2)	12 (2.8)	10 (2.0)	60 (3.1)	
**SES status**							
Low	N (%)	124 (26.1)	48 (11.3)	349 (76.9)	191 (44.3)	712 (39.9)	0.00
Med	N (%)	303 (63.8)	307 (72.1)	98 (21.6)	227 (52.7)	935 (52.4)	
High	N (%)	48 (10.1)	71 (16.7)	7 (1.5)	13 (3.0)	139 (7.8)	
**Obesity Status**							
Normal	N (%)	568 (82.2)	501 (83.5)	535 (88.6)	532 (84.7)	2136 (84.7)	0.02
Overweight	N (%)	74 (10.7)	70 (11.7)	47 (7.8)	67 (10.7)	258 (10.2)	
Obese	N (%)	49 (7.1)	29 (4.8)	22 (3.6)	29 (4.6)	129 (5.1)	

*SES* socio-economic status

^a^*P*-value are derived from Chi squared tests

Overall, in this study, the prevalence of overweight and obesity was equal to 10.2 and 5.1%, respectively, and there was no significant association in the gender groups (prevalence of overweight and obesity was equal to 10.0 and 5.2% for boys and 10.3 and 5.0% for girls, respectively, with *P*-value> 0.05). The results of anthropometric measures in 4 districts are presented in Table 2. Generally, there were statistically significant differences in all anthropometric variables between four groups (*P*-values < 0.01). The results of ANOVA tests revealed that, district 4 and 1 had the highest values of TST, AST, and SST. Means in all anthropometric measures were significantly lower in the third district than those of other districts. Furthermore, prevalence of overweight and obesity in the third district was lower than other regions.

**Table 2:** Anthropometric measurement of individual at district level

	Descriptor	District 1	District 2	District 3	District 4	*P*-value
TST (mm)	Mean (SD)	16.36 (7.33)	15.65 (7.02)	13.08 (5.93)	16.71 (8.25)	0.00
AST (mm)	Mean (SD)	17.49 (9.34)	16.49 (7.78)	14.61 (8.83)	17.57 (9.36)	0.00
SST (mm)	Mean (SD)	16.60 (8.14)	16.10 (7.83)	13.85 (6.71)	17.91 (8.84)	0.00
Height (cm)	Mean (SD)	165.88 (8.69)	167.28 (8.08)	163.62 (8.37)	165.48 (8.60)	0.00
Weight (kg)	Mean (SD)	60.50 (13.99)	60.79 (13.43)	56.47 (12.12)	59.32 (13.97)	0.00
BMI (kg/m^2^)	Mean (SD)	21.86 (4.22)	21.61 (3.85)	21.01 (3.69)	21.59 (4.33)	0.00

*P*-values are derived from ANOVA tests (*p*-value < 0.05 was statistical significant), *SD* standard deviation

Abbreviation: *TST* triceps skinfold thickness, *AST* abdominal skinfold thickness, *SST* subscapular skinfold thickness, *BMI* body mass index

Table 3 illustrates the effect of the covariates on three outcomes in a multivariate multilevel model. As shown in Table 3, being girl, having a family history of obesity, and SES were significantly associated with three anthropometric measures. Although boys had greater mean BMI than girls (mean (SD) of BMI was equal to 21.81 (4.51) for boys and 21.23 (3.49) for girls, respectively, with *P*-value < 0.001), the subcutaneous adipose tissue was thicker in girls than that of boys by over 3, 2 and 2 mm in TST, SST and AST, respectively (for TST: β =3.02, SE = 0.37, *p* < 0.001; for AST: β =2.33, SE = 0.49, *p* < 0.001; for SST: β =2.17, SE = 0.42, *p* < 0.001). Furthermore, subjects who lived in a family with a history of obesity had more fat (for TST: β =2.25, SE = 0.34, *p* < 0.001; for AST: β =3.30, SE = 0.45, *p* < 0.001; for SST: β =3.49, SE = 0.39, *p* < 0.001), than others did. Results of Table 3 also showed that, SES had a significant direct effect on all three anthropometric measures. It was found that, compared to children with low SES, children with high and moderate SES had more TST, AST and SST. The levels of physical activity had a positive relationship with individual outcomes, with significant associations between the moderate to vigorous physical activities. Children with moderate physical activity had higher AST and SST than those with vigorous physical activity by over 2, 2 mm (for AST: β =2.54, SE = 1.40, *p* = 0.051; for SST: β =2.24, SE = 1.20, *p* = 0.047). Compared to children in 14–16 age group, children in age group 16–18 years had less TST (β = − 0.67, SE = 0.34, p = 0.04). Moreover, screen time did not play an important role in three outcome variables.

**Table 3:** associated factors with three anthropometric measures in hierarchical model

Fixed Effects						
	TST		AST		SST	
	**Estimate**	**SE**	**Estimate**	**SE**	**Estimate**	**SE**
**Intercept**	10.49*	1.17	11.11*	1.55	10.60*	1.34
**Sex**						
girls/boy	3.02*	0.37	2.33*	0.49	2.17*	0.42
**Family history of obesity**						
yes/no	2.25*	0.34	3.30*	0.45	3.49*	0.39
**Physical activity**						
mild /vigorous	0.81	1.16	1.67	1.52	1.27	1.32
moderate/ vigorous	1.51	1.04	2.54*	1.40	2.24*	1.20
**SES**						
moderate/low	0.81*	0.39	1.01*	0.52	0.80*	0.45
high/low	1.70*	0.66	2.25*	0.90	2.48*	0.77
**Age groups**						
group2/group1	−0.67*	0.34	−0.09	0.46	0.54	0.39
group3/group1	0.30	1.30	−1.09	1.76	1.75	1.51
**Screen time (min per day)**						
watching TV or video games	0.06	0.08	0.15	0.10	0.11	0.09
**Random Effects**						
	**TST**		**AST**		**SST**	
**Variance**	**Estimate**	**SE**	**Estimate**	**SE**	**Estimate**	**SE**
Child-level	38.85	1.46	70.82	2.66	52.32	1.97
District-level	1.18	0.62	1.63	0.94	1.33	0.74
	**TST, AST**	**SE**	**TST, SST**	**SE**	**AST, SST**	**SE**
**Covariance**						
Child-level	35.85	1.69	32.41	1.47	48.71	2.07
District-level	0.68	0.62	0.27	0.51	1.18	0.76
**Correlation**						
Child-level	0.68	**–**	0.72	–	0.80	–
District-level	0.49	**–**	0.21	–	0.80	–

Abbreviation: ***TST*** triceps skinfold thickness, ***AST*** abdominal skinfold thickness, ***SST*** subscapular skinfold thickness, ***SES*** socio-economic status

Age groups: **group1** [14–16) years, **group2** [16–18) years and **group 3** [18–20] years

*p-value < 0.05

-2loglikelihood statistic with Iterative Generalized Least Squares (IGLS) as an estimation method was obtained as 26,653.0 with 42 estimated parameters in final model, so that compared to the null model (null model is a model having only intercepts with the -2loglikelihood of 47,697.8 with 15 estimated parameters), the deviance was statistically significant (21,044.8 with 27 degree of freedom and *P*-value< 0.001) and given the dramatic reduction in deviance, this model fits the data well.

Overall, the major portion of the total variation in TST (97.1%), AST (97.7%), and SST (97.5%) was found at the child level. Further, at the child level (within-districts), high correlations were obtained between three outcomes (the within-district correlations were obtained as 0.68, 0.72, and 0.80 for the (TST, AST), (TST, SST), and (AST, SST), respectively). Although districts explain a relatively small amount of the total variation of TST (2.9%), AST (2.3%) and SST (2.5%), relatively high correlations between the outcome variables indicated that the districts are properly positioned in the third level of the hierarchy. The results of the correlation between the outcomes showed that, the intra-district correlations were obtained as 0.49, 0.21, and 0.80 for the (TST, AST), (TST, SST), and (AST, SST), respectively.

## Discussion

The present study was an attempt to jointly evaluate the relationships between three body fat measures with a set of covariates in Iranian mid-adolescents within different 4 districts, using a multivariate multilevel analysis. Given the multifactorial nature of childhood obesity which form a hierarchical structure, we analyzed the data through a multilevel model. One of the main finding of this study is the high positive correlations between TST, AST and SST at the child level, suggesting that children with higher TST tend to also have higher AST and SST after adjusting for a set of covariates at the child and district levels. Moreover, positive correlations were also observed between three outcomes at district level. This finding implies that communities play an important role in promotion of adolescent’s health. Therefore, health behaviors associated with childhood obesity are influenced by a combination of behavioral and environmental factors including community, school and family.

The prevalence of childhood obesity has sharply increased from 1990 to 2010 in low- and middle-income countries compared to the developed countries [32], which can have undesirable effects on physical, mental, and psychosocial health in adolescents [33–35]. Studies reported that, the prevalence of overweight and obesity in adolescents varies in different parts of Iran [4, 19, 36]. People, who were living in the same region with the same habits were similar in terms of growth, development, and body shape, which might be due to their lifestyle, dietary patterns, and socio-cultural factors [19, 20]. The results of Table 2 revealed that, there were statistically significant differences between the anthropometric measures with respect to 4 districts. Therefore, the effect of individual level risk factors may vary according to the environment in which one lives.

To the best of our knowledge, limited studies have examined the association between individual factors and adiposity indices across children through multivariate multilevel analysis [20, 24]. Results of multivariate multilevel approach showed that, some risk factors associated with the obesity in adolescents were consistent with those reported in previous researches in Iran [19, 20, 37]. Results of multivariate multilevel analysis indicated a statistically significant association between the sex, family history of obesity, and SES with three anthropometric measures. Sex was positively and highly associated with three outcomes, proving that girls had higher TST, AST, and SST than the boys. However, boys had better growth in terms of height, weight and subsequently in BMI than the girls. These results were in line with the previous studies which reported that, the percentage of subcutaneous adipose tissue was higher in females̓ bodies than that of males due to their sedentary lifestyle, less involvement in vigorous physical activities and less expenditure of energy [7, 16]. Although, an agreement has been proved between BMI and TST in some studies [29, 38], BMI may not be a useful parameter in measuring the subcutaneous body fat of children, because changing the body shape occurs in childhood. Furthermore, it fails to differentiate the fat from the muscle mass and may classify children with large muscle into obese children group [18]. Shriraam et al. explained that, BMI is a crude measure, which does not provide a precise assessment of body density [10].

A positive association was found between family history of obesity and anthropometric measures similar to other studies [20, 39]. Khashayar et al. reported that, the odds of obesity in Iranian students with obese parents were about 2 times greater than the others [19]. Environmental factors such as family lifestyle, eating habits and also becoming obese due to the genetic factors are considered as the subset of family history of obesity, and are the most important reasons influencing the persistence of obesity in adulthood [4, 40, 41]. Therefore, modification of diet, having proper physical activities, and health care in the families could be an effective approach to decrease the risk of childhood and adulthood obesity.

In line with previous studies in Iran [19, 20], our findings showed positive relationships between SES with three outcome variables, especially at high levels, which revealed that higher risk of overweight/obesity is related to the social environment. Bahreynian et al. study reported that the prevalence of overweight was greater in areas with high SES, whereas underweight and short stature were more prevalent in areas with low SES [42]. In the current study, students with higher anthropometric measures were living in families with higher SES, as confirmed in some other studies conducted in Iran and some other countries, in which positive significant associations were found between SES and adiposity among children and adolescents in developing countries [20, 24, 43]. It is noteworthy that, the means of body fat, height, weight, and prevalence of overweight and obesity were lower in the students living in district 3 than other children (Table 2). Only 1.5% of families living in this district had a relatively high SES level and about 77% of them were classified as families with low income, educational and occupational levels. These findings highlight the need for planning to increase the level of awareness in the families in order to improve their lifestyle, nutrition and try to have more physical activities.

Several studies have reported time spent in watching TV or playing video games increased the risk of overweight/obesity in children [20, 24, 44]. Moreover, the results obtained in some studies revealed a negative correlation between inactivity/sedentary behavior and physical activities in children and adolescents [25, 45]. In our study, however, there was no statistically significant association between screen time and mild physical activities with anthropometric measures. The results of Table 2 revealed that, the subjects living in districts one and four were more likely to be at risk of obesity with respect to body fat measures and BMI indices than other groups. Adolescents living in these two districts had more physical activities and also spent more time in watching TV or playing computer games compared to other two groups (Table 1). Watching TV and other sedentary behaviors increases the consumption of the most advertised goods, including sweetened cereals, sweets, salty snacks, and sweetened beverages leading to increased appetite, energy intake, thus affecting the body weight in children [46]. Therefore, it seems that the presence of one behavior may be so strong that it cannot compensate for the presence of the other.

One of the strengths of the study was concerned with the results obtained in the random effects section in Table 3. The outcome variables were correlated at the districts and the subject levels, confirming the appropriateness of classifying the individual and district in the second and third hierarchical levels. The major portion of the total variance in TST (97.1%), AST (97.7%), and SST (97.5%) was found at the child level, meaning that children with higher TST tend to have high AST and SST. Results also highlighted the importance of clustering in assessing the relationships between demographic characteristics and anthropometric measures.

The cross-sectional nature of the study could be considered as a limitation in this study, because, it is not clear how response variables are influenced by the covariates. Further studies could take a prospective and time-based approach to obtain more accurate results. Another limitation is the use of a single self-reported item to assess family history of obesity and it may have introduced a bias and underreporting of subjects. The lack of other predictor variables related to adolescent obesity such as eating habits, biological measures, as well as the selection of the district as the only variable in the third hierarchical level were also regarded as the third limitation of the study.

## Conclusion

The results of multivariate multilevel analysis showed that sex, family history of obesity, and SES were significantly associated with three body fat measures and there were positive correlation between three outcomes at the child and district levels. Furthermore, these indices were more prevalent among the students living in districts 1 and 4 than other two districts. Therefore, it is suggested to develop effective interventions to prevent the effects of individual and environmental undesirable factors on childhood obesity in both family and community levels, especially in these two districts.

## Data Availability

The datasets used and/or analyzed during the current study available from the corresponding author on reasonable request.

## Abbreviations

BMI: Body mass index
ST: Skinfold thickness;
WC: Waist circumference
TST: Triceps skinfold thickness
AST: Abdominal skinfold thickness
SST: Subscapular skinfold thickness
PA: Physical activity
SES: Socio-economic status
IGLS: Iterative generalized least squares
